# Hybridization, missing wild ancestors and the domestication of cultivated diploid bananas

**DOI:** 10.3389/fpls.2022.969220

**Published:** 2022-10-07

**Authors:** Julie Sardos, Catherine Breton, Xavier Perrier, Ines Van den Houwe, Sebastien Carpentier, Janet Paofa, Mathieu Rouard, Nicolas Roux

**Affiliations:** ^1^ Bioversity International, Parc Scientifique Agropolis II, Montpellier, France; ^2^ CIRAD, UMR AGAP Institut, Montpellier, France; ^3^ UMR AGAP Institut, Univ Montpellier, CIRAD, INRAE, Institut Agro, Montpellier, France; ^4^ Bioversity International, International Transit Centre, Leuven, Belgium; ^5^ Papua New Guinea (PNG) National Agricultural Research Institute, Southern Regional Centre, Laloki, Port Moresby, Papua New Guinea

**Keywords:** banana (*Musa* ssp.), domestication, hybridization, missing wild ancestor, *Musa acuminata*, selection, introgression

## Abstract

Hybridization and introgressions are important evolutionary forces in plants. They contribute to the domestication of many species, including understudied clonal crops. Here, we examine their role in the domestication of a clonal crop of outmost importance, banana (*Musa* ssp.). We used genome-wide SNPs generated for 154 diploid banana cultivars and 68 samples of the wild *M. acuminata* to estimate and geo-localize the contribution of the different subspecies of *M. acuminata* to cultivated banana. We further investigated the wild to domesticate transition in New Guinea, an important domestication center. We found high levels of admixture in many cultivars and confirmed the existence of unknown wild ancestors with unequal contributions to cultivated diploid. In New Guinea, cultivated accessions exhibited higher diversity than their direct wild ancestor, the latter recovering from a bottleneck. Introgressions, balancing selection and positive selection were identified as important mechanisms for banana domestication. Our results shed new lights on the radiation of *M. acuminata* subspecies and on how they shaped banana domestication. They point candidate regions of origin for two unknown ancestors and suggest another contributor in New Guinea. This work feed research on the evolution of clonal crops and has direct implications for conservation, collection, and breeding.

## Introduction

Domestication holds a special place in the long trajectory of plants evolution that spans over hundreds of millions of years (Morris et al., 2018). Domesticates have indeed emerged in the last 12.000 years as results of co-evolutionary interactions between plants and human populations (Meyer and Purugganan, 2013). This recent evolutionary history – with regards to plant life on earth - resulted in major phenotypic changes in crops and have fascinated biologists since the early beginning of evolutionary studies (Darwin, 1869). Domesticated plants have thus been widely used for developing and testing evolutionary theories (Ross-Ibarra et al., 2007; Meyer and Purugganan, 2013; Turcotte et al., 2014; Gaut, 2015). In addition, the increasing pressure on environment and on food systems pushed research towards a better understanding of the mechanisms underlying the transition from wild, often inedible, plants to high yielding nutritious crops. Logically, studies focusing on unravelling the origins and trajectories of crop species increased in the last decades.

Hybridization, which in plants is a starting point for hybrid speciation and enables the introduction of adaptive variation through introgressions (Soltis and Soltis, 2009; Abbott et al., 2016), appears to also be an important evolutionary force in domesticates. Advances in genetics and genomics have highlighted the prominent roles of hybridization, and of introgression in the creation of plant domesticates, and in their diversification (reviews in Arnold, 2004; Purugganan, 2019). Some of the most important crops on Earth are indeed hybrids between two or more species, such as wheat (Baidouri et al., 2017), sugarcane (Pompidor et al., 2021) or strawberry (Feng et al., 2021). Introgressions from related species, such as in sunflower (Baute et al., 2015), or from different genepools of same species, such as in maize (Gonzalez-Segovia et al., 2019) and Asian rice (Santos et al., 2019), also have contributed to shape domesticates diversity.

In the global picture of domestication studies, vegetatively propagated crops hold a special place, especially since their evolutionary history was long under-considered. However, many clonal crops, in addition to be of high economic importance, are also critical for food security in many developing countries. This is notably the case of cassava (*Manihot esculenta* Crantz), sweet potato (*Ipomoea batatas* L.), potato (*Solanum tuberosum* L.), yams (*Dioscorea* spp.) or bananas (*Musa* ssp.). The clonal nature of these crops has indeed led to the over-simplified assumption that most of them were resulting from the capture and clonal multiplication of interesting wild genotypes (McKey et al., 2010). However, a few in depth studies provided interesting insights, highlighting for some species the regular occurrence of sexual reproduction despite their vegetative mode of propagation, as in *Ensete ventricosum* (Shigeta, 1996), cassava (Pujol et al., 2005; Sardos et al., 2008) and yams (*Dioscorea* spp.) (Chaïr et al., 2010), or a hybrid nature for other species (Chaïr et al., 2016; Feng et al., 2021; Pompidor et al., 2021). Introgressions, who contributed to local adaptations were also identified, such as in apple (Cornille et al., 2012; Sun et al., 2020), and sometimes revealing complex schemes of crossing, such as in some citrus species (Wu et al., 2018; Ahmed et al., 2019). These studies also confirmed that not only the vegetatively propagated crops were under-studied, but their wild relatives were too (McKey et al., 2010), with the striking example of the greater yam (*D. alata* L.), a species of high importance in Africa and the Pacific, and for which no wild ancestral population was identified yet (Chaïr et al., 2016).

Banana (*Musa* spp.) is a vegetatively propagated crop native to a wide South-East Asia/Oceania region (Simmonds, 1962). It was domesticated from *Musa acuminata* (A genome), a wild species belonging to the monocots, more than 7,000 years ago, likely in New Guinea island (Denham et al., 2003). In this crop, the main traits selected during the wild-to-domesticate transition are parthenocarpy, i.e. the ability to set fruits without the need of prior pollination, and sterility (Simmonds, 1962; Denham et al., 2020). Together, parthenocarpy and sterility ensure the production of edible fleshy fruits that are free of seeds. They also make banana breeding quite challenging, as it is difficult to produce high-quality sterile and parthenocarpic improved varieties through recombination of fertile and non-parthenocarpic parents. Banana breeding is further hampered by the multiple levels of ploidy that can be found in cultivars (diploids, triploids, and tetraploids) and that reduce the number of potential diploid parents for crosses. In such context, the characterization of the diploid germplasm available and the better understanding of the wild-to-domesticate transition is key for the success of breeding schemes.

Cultivated bananas currently encompasses a wide diversity of cultivars of both dessert and cooking types. This diversity includes cultivars of pure *M. acuminata* ancestry and inter-specific hybrids between *M. acuminata* and a few other species (Heslop-Harrison and Schwarzacher, 2007). The simplistic view that interesting genotypes were captured and then conserved clonally does not apply to the domestication of bananas. First, back-crossing was confirmed in the making of interspecific hybrids (Baurens et al., 2019; Cenci et al., 2021). Second, intra-specific hybridization also occurred between different subspecies of *M. acuminata* (Carreel et al., 2002; Perrier et al., 2011), leading sometimes to complex genomic structures organized in sub-genomes mosaics (Martin et al., 2020b), and rising substantial questions on the adaptative traits and selective advantages provided by the introgressions of the different sub-species of *M. acuminata* involved in the setup of cultivars.

*Musa acuminata* is a complex of sub-species that are geographically segregated across a gradient spanning from East India and Sri Lanka to Papua New Guinea, and the northern tip of Queensland in Australia. Subspecies show unique features, morphologically (Simmonds, 1956), genetically (Hippolyte et al., 2012) and at the genome level (Martin et al., 2020a). The New Guinea subspecies *M. acuminata* ssp. *banksii* had a prominent role in the domestication of bananas and is believed to be the genepool at the origin of parthenocarpy (Simmonds, 1956). Four other subspecies are long known to have also contributed to the genetic set up of cultivated bananas: ssp. *burmannica*/*siamea* that is found from southern India and Sri Lanka to Cambodia, ssp. *malaccensis* located in the Malayan peninsula, ssp. *zebrina* in Java and ssp. *errans* in the Philippines (Carreel et al., 2002; Perrier et al., 2011). However, recent studies of small sets of banana cultivars with pure *M. acuminata* ancestries revealed the existence of several ancestral genepools contributing to the setup of banana cultivars and that are missing in the current representation of wild *M. acuminata* diversity (Martin et al., 2020b; Jeensae et al., 2021). Where these undefined genepools originated, how much they contributed to the diversity of banana cultivars, and what were their roles in the domestication and diversification of bananas is not known.

In the present paper, we used SNP markers generated by Restriction site associated DNA sequencing (RADseq; Davey and Blaxter, 2010) to explore the geographical patterns of diversity in a wide set of diploid bananas composed of 226 *M. acuminata* accessions and *M. acuminata* derived diploid cultivars obtained from genebanks and collecting missions. We aimed at (1) determine the diversity, distribution, and geographical radiation of *M. acuminata* in its natural range, (2) assess the contribution of the undefined genepools to the diversity of banana cultivars and identify candidate regions for their origins, and (3) clarify the processes of the transition between wild and domesticated plants by comparing the genetic diversity patterns of wild *M. acuminata* ssp. *banksii* and derived cultivated diploid bananas in New Guinea. This work has impact for *Musa* spp. genetic resources conservation and collection, banana breeding, and evolutionary biology research on vegetatively propagated crops.

## Materials and methods

### Plant materials

A set of 226 diploid banana accessions was selected (**Table 1** **;** **Supplementary Table S1**, **Supplementary Material online**). This set comprised 68 wild accessions belonging to subspecies of *M. acuminata*, 154 related edible diploid cultivars, three accessions of *M. schizocarpa* considered as outgroup and a hybrid between *M. acuminata* ssp. *banksii* and *M. schizocarpa*. These materials were provided by the ITC (170 samples Musa Germplasm Information System (MGIS - https://www.crop-diversity.org/mgis/) (Ruas et al., 2017), the banana collecting mission to the AROB (25 samples) (Sardos et al., 2018), CIRAD (24 samples) (Perrier et al., 2019), collecting missions to Indonesia (4 samples) (Sutanto et al., 2016) and EMBRAPA (3 samples).

**Table 1:** Origins and classification of the sample accessions.

CLASSIFICATION		ORIGIN											
	n	PNG	Hawaii	Philippines	Indonesia	Malaysia	Indo/malaysia	Thailand	Vietnam	Myanmar	India	East Africa	Not recorded
AA (CVs)	*154*	76	0	15	9	12	7	7	3	0	1	22	2
***M*. *acuminata***													
*banksii*	23	20		1	2								
*errans*	1			1									
*zebrina*	6		1		3								2
*sumatrana*	1				1								
*microcarpa*	2				2								
*malaccensis*	12					8		3					1
*truncata*	1					1							
*siamea*	3							3					
*burmannica*	3									3		4	
Other seedy	16				6	2							4
sub-total	***68***	**20**	**1**	**2**	**14**	**11**		**6**	**0**	**3**	**0**	**4**	**7**
***M. schizocarpa***	**3**	3											
***M. ac. x M. schizocarpa***	**1**	1											
	***226***	**100**	**1**	**17**	**23**	**23**	**7**	**13**	**3**	**3**	**1**	**26**	**9**

Bold values correspond to Totals and sub-totals.

### Restriction-site-associated DNA sequencing

DNA from each accession was extracted following a 2X CTAB protocol (modified from Doyle and Doyle, 1990). Library for restriction-site-associated DNA sequencing (RADSeq; Davey & Blaxter, 2010) was built with the PstI restriction enzyme. The 300–500 short-insert libraries were sequenced with 91 bp paired-end reads using Illumina HiSeq2000 (Illumina, San Diego, CA, USA) by BGI Hong Kong. At BGI, the raw data were modified with the following two steps: (1) reads polluted by adapter sequences were deleted; and (2) reads that contained >50% low-quality bases (quality value ≤5) or >10% N bases were removed.

### Read processing and SNP calling

Reads contained in raw FASTQ files (one per sample) were checked using FastQC and then cleaned to remove Illumina adapter sequences and low-quality ends (Phred score > 20) with Cutadapt (Martin, 2011). After trimming, reads inferior to 30 bp were discarded. Reads were then aligned against the *Musa acuminata* genome v2 downloaded on the Banana Genome Hub (Droc et al., 2013) using BWA-MEM (Li and Durbin, 2010). Re-alignment was done with the IndelRealigner module from GATK v4.1 (McKenna et al., 2010). We then followed the GATK pipeline recommended for a non-model organism by adding the recalibration step. It consisted of performing an initial round of SNP calling on the original uncalibrated data, selecting the SNPs with the highest confidence, and then executing a round of base recalibration on the original mapped reads files. The SNP calling was done with the GATK module HaplotypeCaller v4.1 to call SNPs and indels. Considering inter-sample variation, the SNP calling was done on all samples simultaneously. The pipeline used to perform those analyses is available at https://github.com/CathyBreton/Genomic_Evolution.

### Genetic diversity analyses

For the initial set of 226 accessions, SNPs were filtered for missing data (5% as maximum allowed) and MAF (1%), yielding a total of 39,031 bi-allelic SNPs. At this stage, eight accessions were discarded as having more than 15% of missing data.

A dissimilarity matrix was calculated following the Simple-Matching index with a minimum of 70% of common sites between each pair of remaining individuals with DARwin 6 (Perrier and Jacquemoud-Collet, 2006). A weighted Neighbor-Joining (NJ) tree rooted using *M. schizocarpa* as outgroup was then constructed. This first tree allowed identifying cultivated accessions exhibiting identical or nearly identical genotypes and corresponding to duplicates or clonal varieties. To avoid potential bias in further analyses due to genotypes redundancy, we then allowed the presence of a single accession per Genotype Clusters, further reducing the set of accessions to 158 individuals. For each Genotype Clusters, selection of the unique representative kept for further analyses was based on the lowest rate of missing data per accession. For this subset of 158 representative accessions, we retrieved a new set of SNPs with a minor allele frequency of 0.01 (1%) or greater and allowing a maximum of 10% of missing data. With the 66,481 SNPs obtained, DARwin 6 was used to calculate a simple-matching distance matrix. A new weighted neighbour-joining tree rooted on *M. schizocarpa* was then constructed on the set of 158 accessions, eliminating potential distortion due to duplicated accessions.

### Global population structure

Using VCFtools (Danecek et al., 2011), we generated a set of SNPs evenly distributed every 100 kb and not allowing missing data to reflect all chromosomal regions. This set involved 1278 SNPs used to investigate the structure of the 158 accessions of the pruned dataset. We used a Bayesian Markov Chain Monte Carlo (MCMC) approach implemented in the program STRUCTURE v2.3 (Pritchard et al., 2000). The admixture model with the assumption of correlated allele frequencies between groups (Falush et al., 2003) was chosen and 5 replicates of each value of k ranging from 1 to 15 were run with a burn-in-length of 50,000 followed by 150,000 iterations of each chain. The most likely true of the values of k was determined by examining DeltaK, an *ad hoc* quantity related to the second order rate of change of the log probability of data with respect to the number of clusters (Evanno et al., 2005) and plotted using STRUCTURE HARVESTER (Earl and vonHoldt, 2012). STRUCTURE was then run again following the same model for the best values of K identified with 5 replicates each and a burn-in length of 200,000 followed by 800,000 iterations of each chain.

### Tests for introgression in cultivated bananas (AA)

The four taxon Patterson’s D test (Green et al., 2010; Durand et al., 2011) was developed to detect introgressions in closely related taxa. It considers an ancestral “A” allele and a derived “B” allele across the genome of four taxa with a tree topology (((P1,P2),P3),O). Under the hypothesis “without introgression” the two allelic patterns at the tip of the tree, “ABBA” or “BABA”, occur with equal frequency. An excess of “ABBA” or “BABA”, reflected by a D-statistic significantly different from zero, indicates potential gene flow between P2 and P3 or P1 and P3, respectively. Here, we used the derived statistic *f_d_* that is a more conservative estimator of introgression developed for small number of SNPs (Martin et al., 2015) implemented in https://github.com/simonhmartin/genomics_general. A R script allowed the calculation of the P-value based on jackknife for the null hypothesis that *f_d_* is 0. The procedure is available at https://github.com/CathyBreton/Genomic_Introgression_ABBA_BBAA_Test.

Two tests were performed. In the first one, 31 Papuan edible AAs closely related to the Papuan wild *M. acuminata* ssp. *banksii* were tested for introgression by the subspecies originating in SEA. In the second one, 23 edible AA from SEA were tested for introgression by the Papuan *M. acuminata* ssp. *banksii*. Accessions selected as representative for each wild taxon are presented in **Supplementary Tables 2**, **3**.

### Population differentiation between wild and cultivated Papuan bananas

Based on STRUCTURE outputs, we considered a sub-cluster of 31 AA from PNG with a *M. acuminata* ssp. *banksii* genomic background over 90%. This population and its wild counterpart are represented in our samples by 31 and 24 accessions respectively. A set of 238,357 SNPs was retrieved from GIGWA (Sempéré et al., 2019; Rouard et al., 2022) allowing a maximum of 50% of missing data for each of the two populations. Using VCFtools, we first assessed observed heterozygosity (Ho) and inbreeding coefficient (F) for each of these accessions. Then, considering 200 kb windows exhibiting more than one SNPs, we calculated the nucleotide diversity (π) and Tajima’s D for each population. Finally, we calculated weighted Fst between the cultivated accessions and their wild relative, genome-wide and for 200 kb windows along chromosomes. To better understand the nature of selection in the cultivated, we then considered the 1% lowest and highest Tajima’s D values and the 1% greater Fst values.

## Results

### Diversity analysis

**Figure 1:**
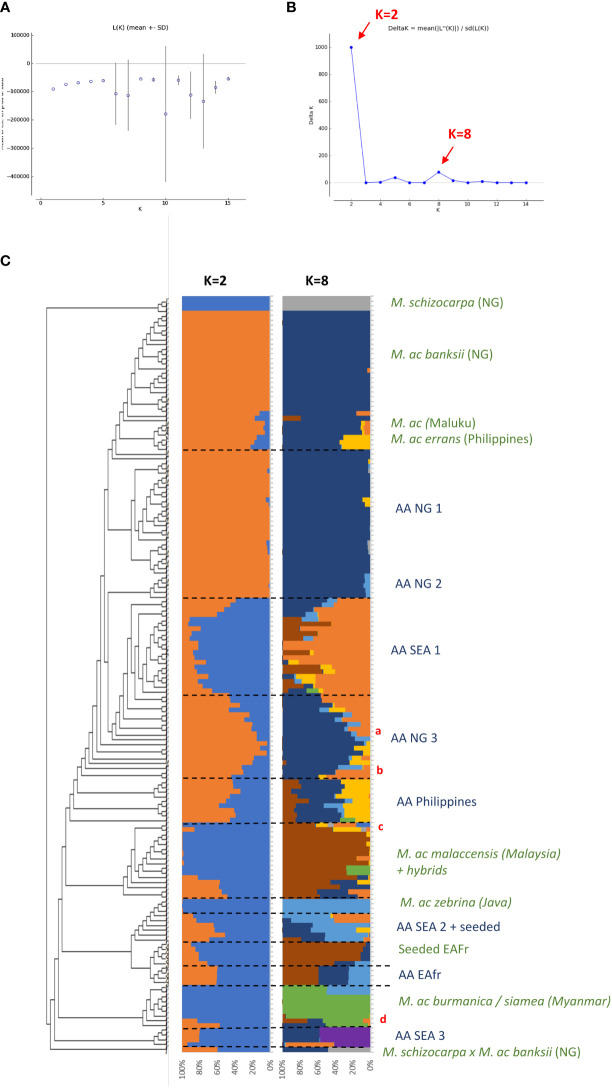
**(A, B)** present the results of the Bayesian clustering of the diploid bananas pruned sample (158 accessions) using STRUCTURE v2.3 (Pritchard et al., 2000) evaluated using STRUCTURE HARVESTER (Earl and vonHoldt, 2012) and based on the lnP(D)/K and DeltaK, respectively. **(C)** Presents the global genetic structure of the pruned sample of 158 genotypes. The cladogram was obtained from the NJ tree constructed with DARwin 6 (Perrier and Jacquemoud-Collet, 2006) on the Simple-Matching distance matrix calculated on 66,481 biallelic SNPs and using FigTree v1.4.3 (Rambaut, 2006-2016) and the R package *ape* (Paradis and Schliep, 2019). Bar plots represent STRUCTURE outputs for K=2 and K=8 as inferred from 1,278 SNPs distributed evenly across the genome, each bar corresponds to a genotype and colours correspond to the detected ancestral genepools. EAfr: East Africa; SEA: South-East Asia; NG: New-Guinea Island; a: ITC0299 ‘Guyod’ from the Philippines, b: ITC0447 ‘Pu-Te Wey’ from Malaysia, c: ITC1701 *M. acuminata* ssp. *sumatrana* from Sumatra and ITC0393 *M. acuminata* ssp. *truncata* from Malaysia, *d*: ITC1761 ‘Matti’ from India and ITC0610 ‘Tuu Gia’ from Vietnam.

The dissimilarity matrix and the NJ Tree obtained on the whole sample set comprising 226 individuals (**Table 1** and **Supplementary Figure 1**) allowed the identification of 158 distinct genotypes including 26 genotype clusters (GC) and 132 unique genotypes (**Supplementary Table 1**). In the NJ tree constructed on this pruned dataset, the subspecies of *M. acuminata*: *banksii*, *malaccensis*, *zebrina* and *burmannica*/*siamea* formed segregated clusters. Three out of the four *M. acuminata* accessions (AMB007, AMB008 and Sup4), collected in Maluku islands (Indonesia, west of NG island), clustered at the margin of ssp. *banksii* while the fourth one AMB004 clustered within ssp. *banksii* from New Guinea. The philippino *M. acuminata* ssp. *errans*, represented by the accessions ‘errans’ and ‘UPLB’ (initially classified as ssp. *banksii*) also clustered at the margin of ssp. *banksii*, along with ‘Borneo’ classified as ssp. *microcarpa* and collected in Borneo. The subspecies *sumatrana* and *truncata* from Sumatra and the Malay Peninsula respectively, clustered with ssp. *malaccensis* while the seeded plants collected in East Africa clustered with ssp. *zebrina* (**Figure 1A**).

Cultivated AA accessions are spread over the different clusters (**Figure 1A**). A first cluster composed only of edible AA accessions from New Guinea island (‘AA NG 1’) is tightly linked to the ssp. *banksii* cluster. A group of accessions from South-East Asia (‘AA SEA 2’), mixed with various seeded hybrids, clusters with ssp. *zebrina*, such as seeded and edible AA bananas from East Africa (‘Seeded EAfr’ and ‘AA EAfr’). Two accessions, ‘Matti’ and ‘Tuu Gia’ from India and Vietnam respectively, clustered with ssp. *burmannica*/*siamea.* The nine accessions from the Philippines form two clusters that are not linked to any wild representative, such as two groups of accessions from South-East Asia. The first one (‘SEA 1’) is located at the center of the tree while the second one (‘SEA 3’), composed of accessions classified as Pisang Jari Buaya and of an accession named ‘Pisang Madu’, clusters at the margin of the tree. Two additional groups of edible AA bananas from New Guinea (‘AA NG2’ and ‘AA NG3’) are spread between the ssp. *banksii* and the South-East Asian subspecies of *M. acuminata*.

### Population structure

**Figure 2:**
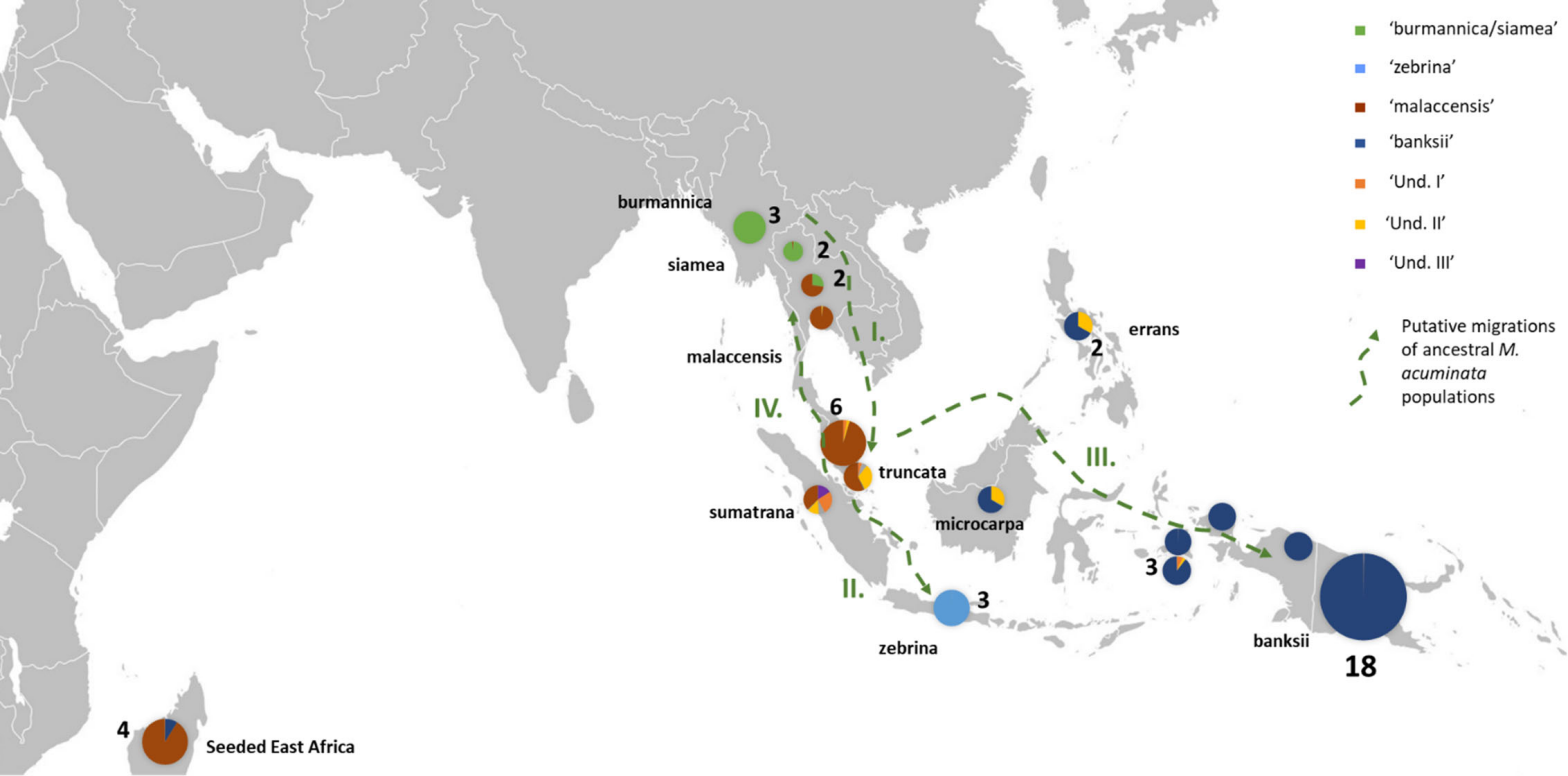
Distribution of the *M. acuminata* accessions of the sample. The pie charts illustrate genomic background (as inferred by STRUCTURE for k=8) and the numbers indicate the number of accessions sharing similar patterns. Putative dispersal roads of ancestral populations are represented by green dashed arrows. I. First dispersion from mainland South-East Asia towards the Malayan Peninsula and Sumatra, followed by II. Dispersal of populations to Java, III. dispersal to Borneo, the Philippines and New Guinea Island and IV. Secondary colonization of mainland South-East Asia. ‘Und.I’, ‘Und. II’ and ‘Und. III’ correspond to genepools for which no parental populations were identified in the samples.

The two best values of k identified by STRUCTURE in the pruned dataset were k=2 and k=8 (**Figures 1B, C**). For k=2, the Bayesian analysis recognized two genepools corresponding roughly to New Guinea island and South-East Asia with a high number of admixed accessions. For k=8, this analysis confirmed discrete genepools for the *M. acuminata* taxa *banksii*, *malaccensis*, *zebrina*, *burmannica*/*siamea* and for *M. schizocarpa*. We noted two accessions from Thailand originally classified as ssp. *malaccensis* that seem to also hold some ssp. *burmannica*/*siamea* signature in their genome (‘Pa (Musore) n°2’ with Q _burmannica/siamea_ = 27,1% and ‘THA018’ with Q _burmannica/siamea_ = 27,3%). In addition, the genomic composition inferred for ‘truncata’ and ‘sumatrana’ accessions – belonging to eponymous subspecies - revealed patchworks of different genepools with a *malaccensis* dominance in both. However, their respective genomic profiles are slightly different (**Figures 1A**, **2**).

**Figure 3:**
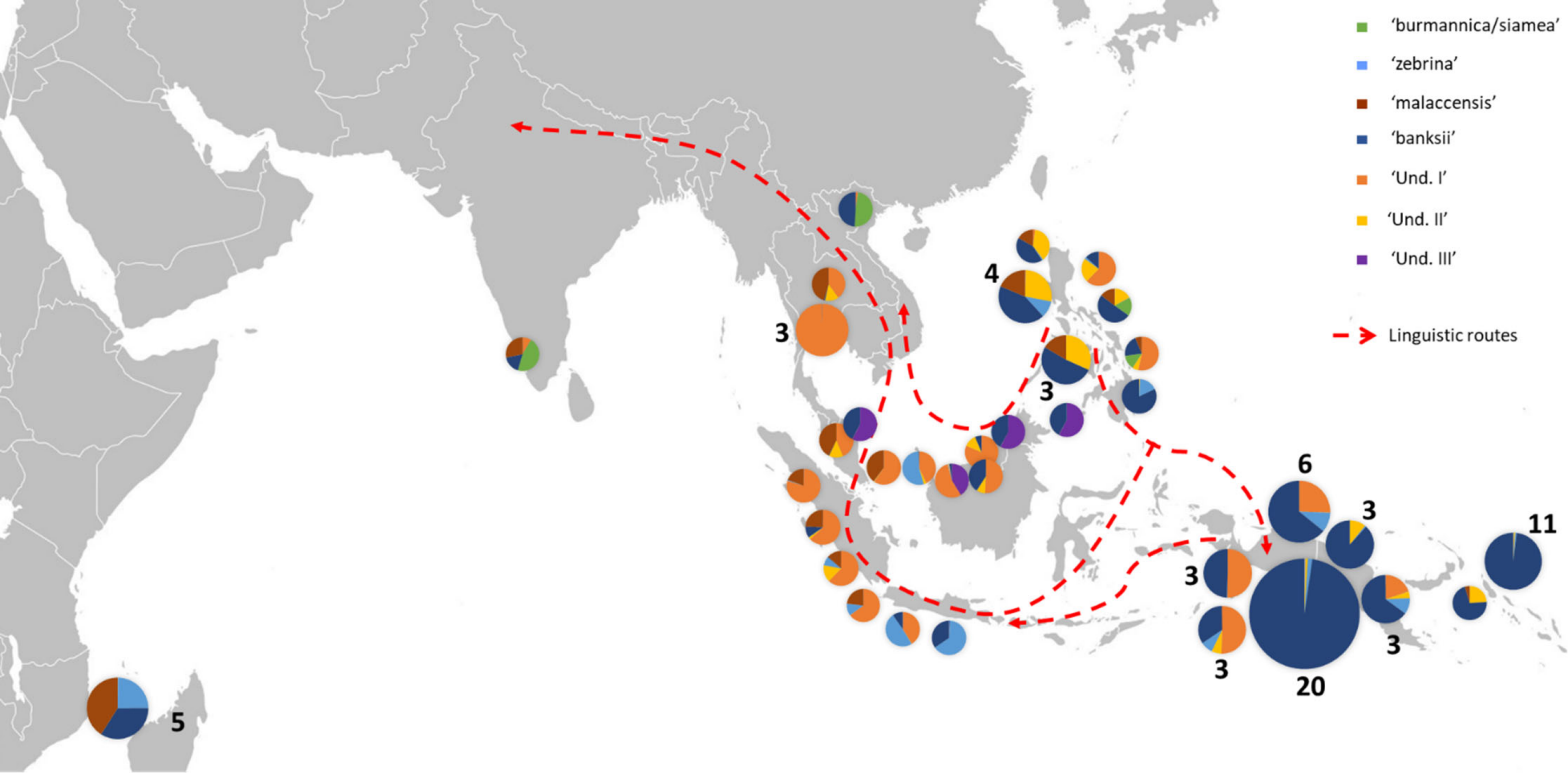
Distribution of cultivated AA accessions of the samples. The pie charts illustrate genomic background (as inferred by STRUCTURE for k=8) and the numbers indicate the number of accessions sharing similar patterns. Dashed red lines show linguistic paths for the words ‘banana’ in the region as inferred by Perrier et al. (2011). The historical movements of these terms reflect ancient spread of *Musa* cultivars in the region.

Three undefined genepools, i.e. for which unadmixed individuals from the source populations are absent in the sample, were detected. The first one (denoted ‘Und-I’) (orange colored in **Figures 1A**, **2**, **3**) was very common in cultivated accessions. Three cultivars from Thailand and clustering within ‘AA SEA1’, are fully assigned (Q_Und-I_ > 90%) to the Und-I genepool (‘Thong Dok Mak’, ‘Kluai Lep Mu Nang’ and ‘Sa’). The ‘Und-I’ alleles were detected in many cultivated accessions from SEA and in a few accessions of ‘AA NG3’. It was also inferred as introgressions in some wild specimens spread in different clusters, such as ‘sumatrana’ (Q_Und-I_ = 26%) and three hybrids collected in Indonesian part of New Guinea in the 1960’s and clustering with ssp. *malaccensis* (‘Higa’ and ‘Hybrid’) and with ssp. *banksii* (‘Waigu’).

The two other undefined genepools were inferred as introgressions. The genepool ‘Und-II’ was detected in the two ssp. *errans* specimen and in the accession ‘Borneo’. These three accessions share similar profiles along with a partially common genetic background with ssp. *banksii*. ‘Und-II’ alleles were also detected in some of the cultivated accessions including the two ‘AA Philippines’ clusters (yellow color in **Figure 1A**). The third undefined genepool ‘Und-III’ was identified in the cluster ‘AA SEA3’ (purple color in **Figure 1A**) in which accessions were collected in the north coast of Borneo and on Sulu, an island located between Borneo and the main Philippines islands (**Figure 3**). The genepool ‘Und-III’ was also inferred as a small introgression in the subspecies *sumatrana*.

The inferred genomic composition of the cultivated AA bananas confirmed the hybrid status of most of them. *Musa acuminata* ssp. *banksii* from NG is a prominent contributor to these edible AAs, including those in South-East Asia, followed by the Javanese ssp. *zebrina*, ssp. *malaccensis* from the Malayan peninsula and then ssp. *burmannica/siamea.* The latter ranges from India to north Thailand and is the subspecies contributing the least to the edible samples. Interestingly the two undefined genepools ‘Und-I’ and ‘Und-II’ are important contributors to cultivated diploid bananas. Finally, we noted in this analysis that the 31 cultivated AA accessions in the clusters ‘AA NG1’ and ‘AA NG2’ were inferred with more than 90% of their genome belonging to ssp. *banksii* (**Figures 1C**, **3**).

### Detection of introgressions

#### Introgressions of South-East Asian subspecies into clusters ‘NG 1’ and ‘NG 2’

To enable testing the 31 cultivated AA accessions from New Guinea with Q_banksii_ > 90% for introgression by one or more of the six SE Asian subspecies of *M. acuminata*, we performed 186 tests. Only Patterson’s D tests for which the dominance of the BBAA pattern over ABBA and BABA were considered robust. Following this criteria, ten combinations which all exhibited BABA > BBAA were excluded. Within the remaining 176 tests, Patterson’s D was statistically significantly negative (Z score < -2) for 70 combinations, showing significant excess of BABA sites over ABBA sites and indicating a highest proximity between ssp. *banksii* and the SEA subspecies tested. At the contrary, for 11 combinations, involving 7 accessions, the D scores obtained were significantly positive (Z score > 2), revealing a significant bias towards ABBA pattern compared with BABA and reflecting possible introgression of given SEA subspecies. We noted that the sub-species *zebrina* was detected in the 7 accessions and was the only introgressing genepool for 4 of them. The subspecies ssp. *burmanica/siamea* was detected in 3 accessions. We noticed however that it was always coupled with suspected introgressions from other sub-species, *zebrina* (3 accessions), *sumatrana* (2 accession) and *malaccensis* (1 accession). For 95 combinations tested, D was not significantly departing from 0 (-2 < z-score < 2), therefore not showing significant differences between the counts of ABBA and BABA sites. Among those tests, nine accessions did not showcase any significant differences in the number of ABBA and BABA sites for any of the six SE Asian subspecies tested, suggesting that they may be truly unadmixed cultivated accessions (**Supplementary Table 2**).

#### Introgressions of subspecies banksii into South-East Asian cultivated AAs

We also performed Patterson’s D tests on 33 cultivated AA accessions originating in South-East Asia to check for their introgression by *M. acuminata* ssp. *banksii*. However, for most of the tests, the count of the different patterns showed topology discordance compared to assumption. That is to say that for eight accessions, both ABBA and BABA counts were dominant over BBAA, that for 18 accessions ABBA counts were dominant over BBAA and for one accession, namely ‘Malaysian Blood’, BABA was dominant over BBAA. For all the six tests for which no topology discordance was identified, statistically significant bias towards ABBA pattern were identified when compared to BABA, suggesting introgression of ssp. *banksii* in the accessions tested (**Supplementary Table 3**).

### Pattern of differentiation between *M. acuminata* ssp. *banksii* and related cultivated AAs in New Guinea

**Figure 4:**
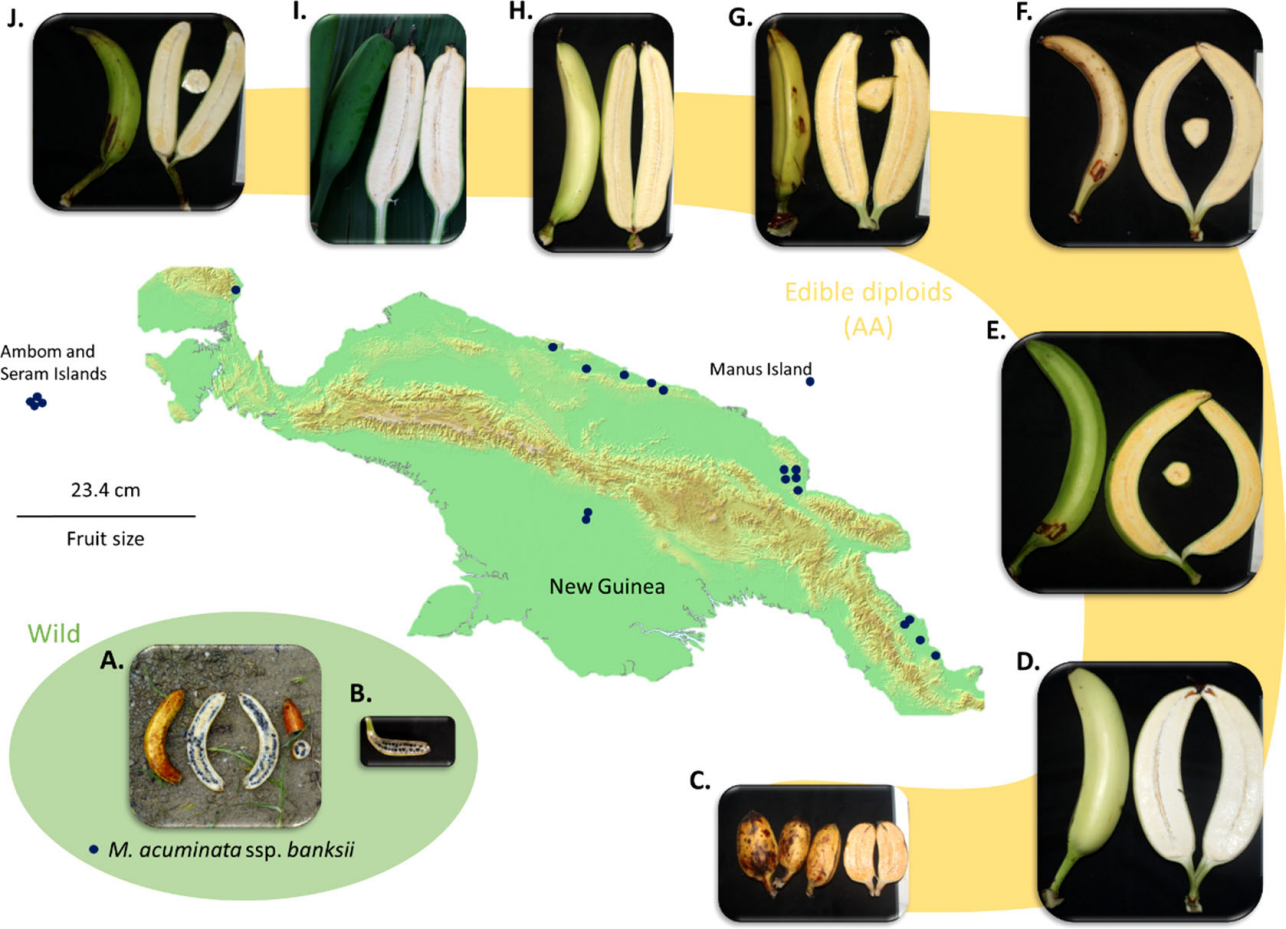
Map of New Guinea Island and examples of fruits of M. *acuminata* ssp. *banksii* **(A, B)** and of closely related cultivated AA accessions from Papua New Guinea **(C)**. AROB003 ‘Mero Mero’, **(D)** AROB034 ‘Nesuri’, **(E)** AROB004 ‘Wiau’, **(F)** AROB035 ‘Talasea’, **(G)** AROB050 ‘Seseve’, **(H)** AROB047 ‘Tobaung’, **(I)** AROB019 ‘Tavilo’, and **(J)** AROB016 ‘Nape’e’). Dark blue dots on the map represent collection locations for accessions within the cluster ‘banksii’ (according to Sharrock, 1988; Sharrock et al., 1988; Sharrock et al., 1989, Sharrock 1989 and Sutanto et al., 2016). Photos: **(A, B)** taken by first author, **(C-J)** courtesy of NARI.

The observed heterozygosity (Ho) of the accessions of the ‘*banksii*’ cluster collected in New Guinea island (**Figure 4**) ranged from 0.02 to 0.12. The four accessions collected in the region of Ambon and Seram, at the west of New Guinea island, had two profiles. The accession AMB004 was similar to the accessions from New Guinea (Ho=0.02) while the three others, AMB007, AMB008 and Sup04, had Ho ranging between 0.30 and 0.34. Inbreeding coefficient (F) confirmed significant excess of homozygous sites for the accessions from New Guinea and AMB004 but not for ‘AMB007’, ‘AMB008’ and ‘Sup04’. It is to be noted that none of the accessions collected in Ambon and Seram, including AMB004, was morphologically classified as belonging to the *banksii* subspecies at collection (Sutanto et al., 2016). In the 31 AA accessions from NG with Q_banksii_ > 0.90, heterozygosity ranged from 0.20 to 0.34 and inbreeding coefficient was reflecting excess of heterozygous sites for 22 accessions (**Table 2**).

**Table 2:** Observed heterozygosity (Ho) and inbreeding coefficient (F) for the accessions of the clusters ‘*banksii*’ and ‘AA NG’.

	ID	Name	N sites	F	Ho
**Cluster *‘banksii’***	AMB004	NA	13538	0.84	0.02
	AMB007	Utang/Biji	15269	-1.09	0.32
	AMB008	Utang/Biji	15247	-0.96	0.30
	ITC0341	Banksii	12862	0.85	0.02
	ITC0428	Higa	9875	0.86	0.02
	ITC0453	Banksii	14587	0.59	0.06
	ITC0606	Hybrid	15136	0.83	0.03
	ITC0616	Hawain 2	13231	0.86	0.02
	ITC0617	Hawain 3	15262	0.84	0.02
	ITC0619	Banksii	14194	0.78	0.03
	ITC0620	Banksii	14896	0.87	0.02
	ITC0621	Banksii	14553	0.76	0.04
	ITC0623	Banksii	14480	0.86	0.02
	ITC0766	Paliama	14706	0.87	0.02
	ITC0806	Banksii	15156	0.88	0.02
	ITC0853	Banksii	14592	0.88	0.02
	ITC0867	Banksii	15236	0.78	0.03
	ITC0879	*Musa* ac. ssp. *banksii*	14872	0.87	0.02
	ITC0885	*Musa* ac. ssp. *banksii*	9800	0.78	0.03
	ITC0897	*Musa* ac. ssp. *banksii*	13061	0.23	0.12
	ITC0937	Banksii	13272	0.86	0.02
	ITC0955	Banksii	14695	0.87	0.02
	ITC1219	Banksii	14867	0.84	0.02
	Sup4	Utang/Biji?	13585	-1.24	0.34
***AA NG (Q_banksii_ > 0.90)***	AROB003	Mero Mero	25904	0.13	0.20
	AROB004	Wiau	25010	-0.15	0.27
	AROB005	Duma	24182	0.09	0.21
	AROB016	Nape’e	25744	-0.03	0.24
	AROB019	Tavilo	26078	-0.33	0.31
	AROB022	Kararu 2	24989	0.06	0.22
	AROB034	Nesuri	26104	-0.04	0.24
	AROB035	Talasea	26122	-0.01	0.24
	AROB047	Tobaung	25856	-0.16	0.27
	AROB049	Nono 2	25954	-0.27	0.30
	AROB050	Sesévé	25898	-0.01	0.23
	ITC0589	Gulum	25181	-0.44	0.34
	ITC0600	Waimara	16022	0.09	0.21
	ITC0770	Navaradam	23952	-0.01	0.24
	ITC0773	Mpiajhap	19885	0.04	0.22
	ITC0778	Gorop	25711	0.08	0.21
	ITC0784	Tamai	25035	-0.02	0.24
	ITC0798	Garunga	19648	-0.20	0.28
	ITC0818	Enar	24672	0.02	0.23
	ITC0847	Hova	16980	0.05	0.22
	ITC0923	Yapu Yapu	24979	-0.16	0.27
	ITC0929	Loibwa	19807	0.09	0.21
	ITC0949	Wiliman	25988	-0.27	0.30
	ITC0984	Yangun Yefan	24766	-0.02	0.24
	ITC1001	Bogia Mun	25866	-0.03	0.24
	ITC1013	Sena	25660	0.09	0.21
	ITC1023	Taoaya	19889	-0.23	0.29
	ITC1206	Spiral	26107	-0.01	0.24
	ITC1220	Yalumia	25961	-0.15	0.27
	ITC1244	Mapua	25848	0.10	0.21
	ITC1245	Papat	25459	-0.48	0.34

**Figure 5:**
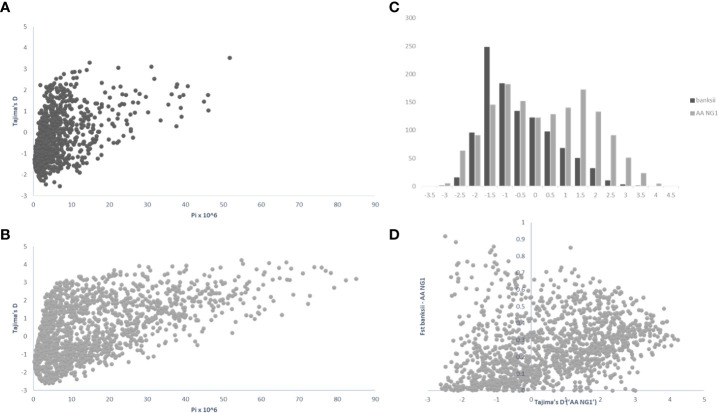
Distribution of pi (π), Tajima’s D and Fst calculated on 200 kb windows along the genomes for the clusters ‘*banksii*’ and ‘AA NG’ (Q_banksii_>90%). Tajima’s D plotted against pi (π) for ‘*banksii*’ **(A)** and ‘AA NG’ **(B)**; comparative distribution of Tajima’s D values **(C)** and Tajima’s D calculated for ‘AA NG’ plotted against Fst between ‘*banksii*’ and ‘AA NG’ **(D)**.

For 24 accessions assigned to the subspecies *banksii*, we identified 1059 windows of 200kb size with more than 1 polymorphic SNP. For each window, Tajima’s D values were plotted against nucleotide diversity (π) (**Figure 5A**). Mean Tajima’s D in these windows was -0.28 (variance = 1.28) and the distribution was skewed towards negative values, reflecting a global excess of low frequency variants (**Figures 5A–C**), that can be interpreted as a signature for a recent expansion of the population after a bottleneck. The 31 cultivated AA diploids selected have a greater diversity as expressed by nucleotide diversity (π) and the highest number of 200kb windows with more than 1 variable SNP (1502). For these edible AA, mean Tajima’s D parameter was 0.50 and the distribution of the values obtained for the windows was somewhat bimodal (variance = 2.47) with the main peak being largely negative, reflecting excess of low frequency variants. The second peak is largely positive, reflecting excess of common variants (**Figures 5B, C**). Windows with Tajima’s D below the 1% lower limit (-2.39) and above the 1% upper limit (3.72) for the 31 AA from NG are presented in **Table 3**.

**Table 3:** Number of SNPs, Pi (π) and Fst between the clusters ‘*banksii*’ and ‘AA NG’ for the windows (200 kb) with the 1% lowest and 1% highest values of Tajima’s D calculated for ‘AA NG’.

		*‘AA NG’*			*‘banksii’*			
Chrom.	Window	SNPs	π	Taj.’s D	SNPs	π	Taj.’s D	Fst
chr01	11000000	40	73.95	3.85	27	12.25	-1.78	0.31
chr01	11200000	24	49.32	3.84	11	6.30	-1.33	0.32
chr01	11400000	15	30.28	3.85	17	9.08	-1.30	0.26
chr02	23800000	32	66.65	4.13	26	14.08	-1.46	0.31
chr02	24000000	26	54.80	4.24	29	11.57	-1.81	0.30
chr02	24200000	30	55.18	4.05	31	16.98	-1.24	0.27
chr02	24400000	25	47.13	3.73	15	6.53	-1.81	0.36
chr02	24600000	29	62.04	4.08	29	12.24	-1.91	0.33
chr02	25000000	18	36.61	3.90	1	0.37	0.00	0.33
chr06	1800000	35	67.84	3.79	2	0.60	-1.21	0.40
chr10	21800000	33	64.73	3.81	3	6.15	1.65	0.40
chr10	28400000	22	40.21	3.72	3	1.66	-0.71	0.35
chr10	28600000	20	40.28	3.93	3	1.88	-0.50	0.33
chr10	29600000	30	58.82	3.74	2	1.84	-0.22	0.29
chr11	1	40	75.93	3.84	2	2.30	0.15	0.49
chr11	4800000	14	27.89	3.93	0	0.00	0.00	0.41
chr01	6200000	33	6.95	-2.48	5	3.29	-0.75	0.14
chr02	19800000	21	3.29	-2.58	1	0.24	0.00	-0.01
chr03	29600000	29	5.84	-2.40	2	1.14	-0.61	0.08
chr03	34400000	27	5.53	-2.45	2	0.79	-1.00	0.01
chr04	3200000	15	2.25	-2.48	2	1.67	-0.38	0.02
chr04	3400000	17	2.88	-2.48	3	1.30	-1.25	0.00
*chr04*	*5000000*	*22*	*4.20*	*-2.48*	*3*	*4.92*	*1.42*	*0.92*
chr06	9800000	21	3.15	-2.60	4	1.49	-1.18	0.00
chr06	10000000	26	4.94	-2.46	3	3.50	0.62	0.03
chr06	10200000	26	4.92	-2.49	4	3.10	-0.46	0.03
chr06	10400000	29	5.19	-2.59	4	2.93	-0.74	0.01
chr06	11600000	26	4.16	-2.62	5	2.74	-0.55	0.04
chr06	17400000	13	1.93	-2.42	0	0.00	0.00	-0.01
chr08	7200000	20	3.47	-2.52	2	0.45	-1.46	-0.01
chr08	38000000	45	11.40	-2.39	4	3.65	-0.32	0.06
chr11	27800000	16	2.89	-2.43	1	0.23	0.00	-0.01

Whole genome Fst calculated between ssp. *banksii* and the cultivated AA from NG was 0.30. Considering 200 kb windows exhibiting more than 1 polymorphic SNP, the highest Fst value was 0.92 calculated on 25 SNPs on chromosome 4 (bin start 5.000.000). Tajima’s D value for this genomic region was also among the 1% lowest Tajima’s D values calculated along the genome. Given that Tajima’s D values in the 31 AA from NG plotted against Fst shows that most windows with negative Tajima’s D exhibits also low Fst (**Figure 5D** and **Supplementary Table 4**), this genomic region on chromosome 4 is likely under strong selection.

## Discussion

This study enables proposing hypotheses on the radiation of *M. acuminata* in South-East Asia and New Guinea and highlighting probable geographic regions of origins for the undefined genepools. Finally, by focusing on New Guinea Island we provide new insights on the wild-to-domesticates transition in the banana crop.

### Secondary centers of radiation for *M. acuminata* are in Sumatra and the Malay peninsula

The geographic distribution of the diversity of the *M. acuminata* subspecies as detected in this study enables the proposition of a refined dispersal scenario. *Musa acuminata* arose about 10 million years ago, probably in the northern Indo-Burma region (Janssens et al., 2016). Phylogenetic and phylogeographic studies performed on different markers obtained discordant results on its diversification and its dispersal at the intraspecific level (Janssens et al., 2016; Rouard et al., 2018; Fu et al., 2022). Whole genome sequences of four *M. acuminata* subspecies, ssp. *burmannica*, *zebrina*, *banksii* and *malaccensis*, showed ssp. *burmannica* as the first of the sub-species to have diverged in the species tree (Rouard et al., 2018). This finding suggested that this early divergence occurred in the indo-burmese area, consistently with the inferred origin of *M. acuminata* in this region (Janssens et al., 2016). These genomic results also suggested secondary diversification and dispersal of the ancestors of the others *M. acuminata* subspecies through the Malayan peninsula to Java, and then to New Guinea island and back to the southern Indo-Malayan region (Rouard et al., 2018). Using chloroplast sequences, Fu et al. (2022) confirmed the early emergence of ssp. *burmannica* from the Indo-Burma region, as well as dispersal and emergence of the other subspecies towards Island South-East Asia and New Guinea but with a slightly different pattern. At the east, ssp. *banksii* emerged in New Guinea on the one hand while ssp. *zebrina* and spp. *microcarpa* appeared on Java and Borneo on the other hand. At the west, ssp. *malaccensis* and ssp. *truncata* diverged on the Malayan peninsula.

In this study, we confirmed the anticipated background described for the wild samples (Carreel et al., 1994; Perrier et al., 2011; Sardos et al., 2016; Christelova et al., 2017; Martin et al., 2020b) but we detected for the first time admixed profiles for ssp. *truncata* and for ssp. *sumatrana*. Although we cannot exclude that their under-representation, only one individual of each present in the set, influences the result, we hypothesize that these patterns result from shared ancestries with the different taxa inferred as introgressions, including the more distant *M. schizocarpa*. These shared ancestries are consistent with the proposed role of the Malayan peninsula and Sumatra as centers of secondary diversification and radiation for *Musa* section bananas (Janssens et al., 2016). Interestingly, ssp. *microcarpa* in Borneo and ssp. *errans* in the Philippines, which are both tightly linked to *M. acuminata* ssp. *banksii* in New Guinea, also share common ancestries with ssp. *sumatrana* and *truncata*. This pattern pleads for a dispersal road leading to ssp. *banksii* through Borneo and the Philippines rather than through Java. It also suggests independent subspeciation of ssp. *zebrina* in Java. Both ssp. *sumatrana* and ssp. *truncata* also share ancestry with ssp. *malaccensis*. It argues for the recolonization of the north of the peninsula from the secondary radiation center, as inferred from Rouard et al. (2018). Introgressions of ssp. *burmannica/siamea* into several *M. acuminata* ssp. *malaccensis* samples from Thailand confirm genetic contacts between both genepools in the region (Rouard et al., 2018; Martin et al., 2020b). Therefore, based on these results, we propose a refined dispersal scenario for *M. acuminata* with important secondary centers of radiation in Sumatra and the Malay peninsula from which three dispersion roads are inferred. The first one goes to Java, the second one passes through Borneo and the Philippines towards New Guinea and the third one goes back up to mainland South-East Asia (**Figure 2**).

### Evidence for undefined ancestral genepools in cultivated diploids and their presumed origins

In congruence with Perrier et al. (2011), the genomic constitutions inferred for cultivated AA diploids showed high levels of admixture with patterns that follow the routes of linguistic diffusion in both directions. At the extremes of the species range, the eastern ssp. *banksii* signature was identified in the sole AA specimen from India and as far as in East Africa, while the Myanmar ssp. *burmannica*/*siamea* at the west, which contributes the least to cultivated diploids, was detected as introgressions in accessions from Vietnam and the Philippines (**Figures 1A**, **3**). Our analysis also revealed three genepools for which no reference wild accessions were identified. It is consistent with recent findings of Martin et al. (2020b), who identified two cryptic ancestor populations co-existing within an accession called ‘Pisang Madu’, and of Jeensae et al. (2021), who reported a new genepool in some cultivated bananas. With the presence in our set of the clones ‘Pisang Madu’ and ‘Pisang Mas’, common to each study respectively, we assume ‘Und I’ and ‘Und III’ as the two cryptic ancestors inferred by Martin et al. (2020b) and ‘Und I’ as the unknown genepool discovered by Jeensae et al. (2021).

Our analysis applied to a wide set of accessions highlights the important contribution of the genepool ‘Und I’ to cultivated AA bananas. This genepool was indeed inferred in accessions of all origins, with the noticeable exception of the East Africa region. Based on linguistic and genetic evidence, the early origin of the East Africa bananas was pointed in the southeastern part of Indonesia, in a region between Java, Sulawesi and the western tip of New Guinea island (Perrier et al., 2011; Perrier et al., 2019), which could suggest that ‘Und I’ was not present in this region at this early time. Since the genepool ‘Und I’ is prominent in Thailand, Malaysia and Indonesia, we hypothesize its origin around the Gulf of Thailand and the west of the South China Sea, probably in mainland Southeast Asia.

On the contrary and according to our results, the genepool ‘Und III’ is rare in cultivated bananas and was only found in ‘Pisang Madu’ and in the accessions classified as belonging to the ‘Pisang Jari Buaya’ subgroup. The collection sites of these accessions plead for a potential origin of ‘Und-III’ in Island South-East Asia rather than on the continent, maybe in a region between north Borneo and the Philippines, but further investigation should be conducted (**Figure 3**).

Finally, the genepool ‘Und II’ inferred in our analysis appeared to be an important contributor to cultivated diploids. It was notably found as introgressions in nearly all cultivated accessions from the Philippines and in landraces from South-East Asia and from New Guinea (**Figures 1A**, **3**). The genepool ‘Und. II’ was also inferred as introgressions in ssp. *sumatrana*, *truncata*, *errans* and in the ‘Borneo’ accession, classified as belonging to ssp. *microcarpa* (**Figure 2**). We therefore interpret it as a signature of shared ancestry between these wild accessions, as well as with the cultivated diploids affected. However, as wild populations of *M. acuminata* in Borneo were reported morphologically heterogenous (Häkkinen and Langhe, 2001), we cannot exclude the occurrence of an ancestral population corresponding to ‘Und. II’ on this island.

### From seeded to edible bananas on New Guinea island

As confirmed in this study, *M. acuminata* ssp. *banksii* is a major contributor to cultivated bananas. By comparing a population of 31 closely related cultivated AA landraces from New Guinea to the sample of 24 *M. acuminata* ssp. *banksii*, we aimed at understanding the wild-to-cultivated transition. Unexpectedly, the subset of cultivated diploid AAs revealed higher levels of diversity than its wild relative, as expressed by pi and observed heterozygosity (**Figure 5**). It differs substantially from the standard scenario in which domestication is expected to induce a loss of genetic diversity (Meyer and Purugganan, 2013). Possible explanations for such pattern are multiple and non-exclusive: i) it could result from an insufficient sampling of the wild population, or from the loss of wild genepools after domestication; ii) it may reflect hybridization between genetically distant genepools at the origin of the cultivated population; iii) the accumulation of somatic mutations, a common phenomenon in clonally propagated crops, can also contribute to higher levels of diversity (Miller and Gross, 2011).

Regarding our sampling, *M. acuminata* ssp. *banksii* exhibited very low levels of diversity and a global excess of rare polymorphism as expressed by the distribution of Tajima’s D values. These results point towards a population under expansion after a bottleneck. This bottleneck, reflecting a drastic population reduction, could have been induced by different factors. First, *M. acuminata* ssp. *banksii* displays hermaphrodite flowers causing dominant selfing (Simmonds, 1956; Kallow et al., 2021), an adaptative trait that induce bottlenecks when it emerges in limited numbers of individuals (Foxe et al., 2009; Guo et al., 2009). Second, the last glacial period during which the climate was cooler and drier (Bowler et al., 1976; Hope et al., 2004) likely induced conditions less favourable for *M. acuminata* on the island. Third, early human activities have heavily impacted flora and fauna in this region (Fairbairn et al., 2006) and could have included the wild banana populations. Therefore, if first banana domesticates were extracted from the wild prior the bottleneck, their parental population(s) might have disappeared nowadays. Additionally, we cannot rule out a sampling effect to explain these results as most of the *M. acuminata* ssp. *banksii* accessions studied here were collected in the lowlands of Papua New Guinea (**Figure 4**), so we possibly captured only a portion of the diversity within the taxa. Specimens of *M. acuminata* ssp. *banksii* were also observed at a higher altitude (Eyland et al., 2021) and another genepool is suspected in Indonesian New Guinea (Simmonds, 1956; Argent, 1976), but these regions could not be explored.

Considering hybridization, this is not fully resolved. The Bayesian analysis run by STRUCTURE suggested *M. acuminata* ssp. *banksii* as unique ancestor for this set of cultivated accessions. The four taxa Patterson’s D test that was then run to refine these results revealed introgressions by South-East Asian *M. acuminata* subspecies in seven of the 31 cultivated banana of this set. These results suggest that Patterson’s D test is more sensitive than Bayesian clustering in detecting geneflow. However, it can only be run with taxa that are present in the setting. Therefore, we cannot totally rule out that genetically distant population - absent from the sampling - introgressed cultivated diploids from New Guinea.

Finally, the accumulation of somatic mutations through vegetative propagation creates diversity and increases heterozygosity in clonal crops (McKey et al., 2010). These mutations, as soon as they are not deleterious for the crop, occur and accumulate independently on both haplotypes. As a result, it creates rare diversity that, in a sterile crop, can only be transmitted to clonally derived landraces. Since duplicates and clonemates were removed from the set at the first step of the analysis, signatures of such accumulations can be found in higher heterozygosity levels and negative values of Tajima’s D (**Figure 5**) (further discussed below). However, they cannot explain all the diversity observed in the cultivated set.

The higher levels of diversity observed in the cultivated samples are due to a combination of these factors. Mutations have accumulated through vegetative propagation, increasing heterozygosity and rare alleles. It is also likely that a different wild population, extinct or not, contributed to the genetic make-up of these cultivated diploids.

Besides, in domesticated plants, selection is expected to induce either an excess of low frequency polymorphism due to post-domestication’s bottleneck expansion, as in selfing chickpea (Varshney et al., 2019), or a drop in rare alleles frequencies due to recent selection, such as in clonal African yams (Akakpo et al., 2017). In sugarcane, another clonal crop domesticated in New Guinea, coding regions of the genome were found with higher diversity and with moderate Fst values when comparing the cultivated to the wild. Authors suggested balancing selection and accumulation of mutations were jointly responsible for such pattern (Arro et al., 2016). The signal is not as clear in the edible AAs from New Guinea. The results, including higher diversity and the peak of positive values of Tajima’s D coupled to moderate Fst (**Figure 5**), suggest balancing selection at work. Balancing selection would also explain the higher heterozygosity identified in the cultivated samples. Farming practices could be responsible for such selection. In cassava, for example, farmers unconsciously favour heterozygous, more vigorous, plants that are then clonally propagated (Pujol et al., 2005). However, such as in grape vine, another clonal crop (Houel et al., 2010), balancing selection does not act alone in banana. We identified on chromosome 4 a genomic region that cumulates low Tajima’s D and high Fst values (**Table 3**; **Figure 5**). It strongly suggests positive selection and the fixation of an allele advantageous for the cultivated population. A gene of interest linked to domestication might be located in this region and should be further investigated.

To conclude, hybridization and introgressions have played a major role in the creation of banana domesticates. Undefined genepools contributed massively to the creation of the cultivated bananas of the sampling. In New Guinea, where cultivated diploids are tightly linked to the local wild relative *M. acuminata* ssp. *banksii*, a few introgressions were detected and an uncharacterized genepool is also suspected. The domestication and the diversification of banana therefore result from processes much more complex than expected for a clonal crop where, in theory, advantageous individuals are extracted from the wild and then maintained clonally (McKey et al., 2010).

This study revealed gaps in the knowledge of wild banana genepools that call for future actions. Further explorations of wild bananas species and populations are needed, in particular in the suggested areas of origins of the undefined ancestral genepools. The characterization of the entire wild banana genepool is a prerequisite to understand the diversification history of wild *Musa* species, including *M. acuminata*, and to develop efficient conservation plans for the taxa. It is also a key element for the resolution of the intra-specific hybridization patterns of cultivated bananas. Coupled to the investigation of the correlation between the different wild ancestors’ contributions to cultivars and selected agronomic traits, it will also enable the design of targeted and informed breeding strategies. Finally, with the latest availability of genomic resources and while clonal crops were understudied in the past, it may well be that cultivated bananas offer a unique ground for studying the evolutionary effects of hybridization.

## Data Availability Statement

The datasets presented in this study can be found in online repositories. The names of the repository/repositories and accession number(s) can be found below: https://www.ncbi.nlm.nih.gov/,PRJNA450532.

## Author contributions

JS designed the study. JS and JP performed field collections in PNG and IH provided genebank samples. JS and MR coordinated genotyping experiments. JS and CB performed analyses with inputs from MR, XP and SC. NR supervised the study and acquired funding. JS, CB and MR wrote the manuscript with inputs and review from all authors. All authors approved the final manuscript.

## Funding

This work was financially supported by CGIAR Fund, in particular the CGIAR Research Program, Roots, Tubers and Bananas and the Genebank Platform.

## Acknowledgments

We thank BGI for their technical assistance and services for the RAD sequencing. This work was technically supported by the CIRAD - UMR AGAP HPC Data Centre of the South Green Bioinformatics platform (https://www.southgreen.fr/). We also acknowledge Robert Miller (University of Brasilia), Agus Sutanto (ICHORD), Jeff Daniells (Queensland DAF), Jaroslav Dolezel, Eva Hribova and Pavla Christelova (Institute of Experimental Botany) for providing some of the plant materials and DNA. Thank you also to Rachel Chase (Alliance of Bioversity and CIAT) for editing the manuscript. Finally, we acknowledge all contributors to the International *Musa* Germplasm Transit Centre (ITC). The ITC materials of this study, and more, are available for distribution under the strict terms of the International Treaty on Plant Genetic Resources for Food and Agriculture *via* MGIS (https://www.crop-diversity.org/mgis/).

## Conflict of interest

The authors declare that the research was conducted in the absence of any commercial or financial relationships that could be construed as a potential conflict of interest.

## Publisher’s note

All claims expressed in this article are solely those of the authors and do not necessarily represent those of their affiliated organizations, or those of the publisher, the editors and the reviewers. Any product that may be evaluated in this article, or claim that may be made by its manufacturer, is not guaranteed or endorsed by the publisher.

## References

[B1] Abbott R. J. Barton N. H. Good J. M. (2016). Genomics of hybridization and its evolutionary consequences. Mol. Ecol. 25, 2325–2332. doi: 10.1111/mec.13685 27145128

[B2] Ahmed D. Comte A. Curk F. Costantino G. Francois L. Dereeper A. . (2019). Genotyping by sequencing can reveal the complex mosaic genomes in gene pools resulting from reticulate evolution: A case study in diploid and polyploid citrus. Ann. Bot. 123, 1–21. doi: 10.1093/aob/mcz029 30924905 PMC6612944

[B3] Akakpo R. Scarcelli N. Chaïr H. Dansi A. Djedatin G. Thuillet A.-C. . (2017). Molecular basis of African yam domestication: Analyses of selection point to root development, starch biosynthesis, and photosynthesis related genes. BMC Genomics 18 (1), 782. doi: 10.1186/s12864-017-4143-2 29025393 PMC5639766

[B4] Argent G. C. G. (1976)Wild bananas of Papua new Guinea. In: Notes from the royal botanic garden, Edinburgh. Available at: https://agris.fao.org/agris-search/search.do?recordID=US201302495245 (Accessed January 14, 2021).

[B5] Arnold M. L. (2004). Natural hybridization and the evolution of domesticated, pest and disease organisms. Mol. Ecol. 13, 997–1007. doi: 10.1111/j.1365-294X.2004.02145.x 15078439

[B6] Arro J. Park J.-W. Wai C. M. VanBuren R. Pan Y.-B. Nagai C. . (2016). Balancing selection contributed to domestication of autopolyploid sugarcane (Saccharum officinarum l.). Euphytica 209, 477–493. doi: 10.1007/s10681-016-1672-8

[B7] Baidouri M. E. Murat F. Veyssiere M. Molinier M. Flores R. Burlot L. . (2017). Reconciling the evolutionary origin of bread wheat (Triticum aestivum). New Phytol. 213, 1477–1486. doi: 10.1111/nph.14113 27551821

[B8] Baurens F.-C. Martin G. Hervouet C. Salmon F. Yohomé D. Ricci S. . (2019). Recombination and Large structural variations shape interspecific edible bananas genomes. Mol. Biol. Evol. 36, 97–111. doi: 10.1093/molbev/msy199 30403808 PMC6340459

[B9] Baute G. J. Kane N. C. Grassa C. J. Lai Z. Rieseberg L. H. (2015). Genome scans reveal candidate domestication and improvement genes in cultivated sunflower, as well as post-domestication introgression with wild relatives. New Phytol. 206, 830–838. doi: 10.1111/nph.13255 25641359

[B10] Bowler J. M. Hope G. S. Jennings J. N. Singh G. Walker D. (1976). Late quaternary climates of Australia and new Guinea. Quaternary Res. 6, 359–394. doi: 10.1016/0033-5894(67)90003-8

[B11] Carreel F. Fauré S. González De León D. Lagoda P. Perrier X. Bakry F. . (1994). Evaluation de la diversité génétique chez les bananiers diploïdes (Musa sp). Genet. Selection Evol. 26, 125s–136s.

[B12] Carreel F. Gonzalez de Leon D. Lagoda P. Lanaud C. Jenny C. Horry J. P. . (2002). Ascertaining maternal and paternal lineage within musa by chloroplast and mitochondrial DNA RFLP analyses. Genome 45, 679–692. doi: 10.1139/g02-033 12175071

[B13] Cenci A. Sardos J. Hueber Y. Martin G. Breton C. Roux N. . (2021). Unravelling the complex story of intergenomic recombination in ABB allotriploid bananas. Ann. Bot. 127, 7–20. doi: 10.1093/aob/mcaa032 32104882 PMC7750727

[B14] Chaïr H. Cornet D. Deu M. Baco M. N. Agbangla A. Duval M. F. . (2010). Impact of farmer selection on yam genetic diversity. Conserv. Genet. 11, 2255–2265. doi: 10.1007/s10592-010-0110-z

[B15] Chaïr H. Sardos J. Supply A. Mournet P. Malapa R. Lebot V. (2016). Plastid phylogenetics of Oceania yams (Dioscorea spp., dioscoreaceae) reveals natural interspecific hybridization of the greater yam (D. alata). Botanical J. Linn. Soc. 180, 319–333. doi: 10.1111/boj.12374

[B16] Christelova P. Langhe E. Hribova E. Cizkova J. Sardos J. Husakova M. . (2017) Molecular and cytological characterization of the global musa germplasm collection provides insights into the treasure of banana diversity. Available at: http://biblio1.iita.org/handle/20.500.12478/1488 (Accessed January 14, 2021).

[B17] Cornille A. Gladieux P. Smulders M. J. M. Roldán-Ruiz I. Laurens F. Cam B. L. . (2012). New insight into the history of domesticated apple: Secondary contribution of the European wild apple to the genome of cultivated varieties. PloS Genet. 8, e1002703. doi: 10.1371/journal.pgen.1002703 22589740 PMC3349737

[B18] Danecek P. Auton A. Abecasis G. Albers C. A. Banks E. DePristo M. A. . (2011). The variant call format and VCFtools. Bioinformatics 27, 2156–2158. doi: 10.1093/bioinformatics/btr330 21653522 PMC3137218

[B19] Darwin C. (1869) The variation of animals and plants under domestication (London). Available at: http://darwin-online.org.uk/content/frameset?itemID=F880.1&viewtype=text&pageseq=1 (Accessed August 2, 2021). John Murray.

[B20] Davey J. W. Blaxter M. L. (2010). RADSeq: Next-generation population genetics. Brief Funct. Genomics 9, 416–423. doi: 10.1093/bfgp/elq031 21266344 PMC3080771

[B21] Denham T. Barton H. Castillo C. Crowther A. Dotte-Sarout E. Florin S. A. . (2020). The domestication syndrome in vegetatively propagated field crops. Ann. Bot. 125, 581–597. doi: 10.1093/aob/mcz212 31903489 PMC7102979

[B22] Denham T. P. Haberle S. G. Lentfer C. Fullagar R. Field J. Therin M. . (2003). Origins of agriculture at kuk swamp in the highlands of new Guinea. Science 301, 189–193. doi: 10.1126/science.1085255 12817084

[B23] Doyle J. J. Doyle J. L. (1990). A rapid total DNA preparation procedure for fresh plant tissue. Focus 12, 13–15 .

[B24] Droc G. Larivière D. Guignon V. Yahiaoui N. This D. Garsmeur O. . (2013). The banana genome hub. Database (Oxford) 2013, bat035. doi: 10.1093/database/bat035 23707967 PMC3662865

[B25] Durand E. Y. Patterson N. Reich D. Slatkin M. (2011). Testing for ancient admixture between closely related populations. Mol. Biol. Evol. 28, 2239–2252. doi: 10.1093/molbev/msr048 21325092 PMC3144383

[B26] Earl D. A. vonHoldt B. M. (2012). STRUCTURE HARVESTER: A website and program for visualizing STRUCTURE output and implementing the evanno method. Conserv. Genet. Resour. 4, 359–361. doi: 10.1007/s12686-011-9548-7

[B27] Evanno G. Regnaut S. Goudet J. (2005). Detecting the number of clusters of individuals using the software STRUCTURE: A simulation study. Mol. Ecol. 14, 2611–2620. doi: 10.1111/j.1365-294X.2005.02553.x 15969739

[B28] Eyland D. Breton C. Sardos J. Kallow S. Panis B. Swennen R. . (2021). Filling the gaps in gene banks: Collecting, characterizing, and phenotyping wild banana relatives of Papua new Guinea. Crop Sci. 61, 137–149. doi: 10.1002/csc2.20320

[B29] Fairbairn A. S. Hope G. S. Summerhayes G. R. (2006). Pleistocene occupation of new guinea’s highland and subalpine environments. World Archaeology 38, 371–386. doi: 10.1080/00438240600813293

[B30] Falush D. Stephens M. Pritchard J. K. (2003). Inference of population structure using multilocus genotype data: Linked loci and correlated allele frequencies. Genetics 164, 1567–1587. 12930761 10.1093/genetics/164.4.1567 PMC1462648

[B31] Feng C. Wang J. Harris A. J. Folta K. M. Zhao M. Kang M. (2021). Tracing the diploid ancestry of the cultivated octoploid strawberry. Mol. Biol. Evol. 38, 478–485. doi: 10.1093/molbev/msaa238 32941604 PMC7826170

[B32] Foxe J. P. Slotte T. Stahl E. A. Neuffer B. Hurka H. Wright S. I. (2009). Recent speciation associated with the evolution of selfing in Capsella. PNAS 106, 5241–5245. 19228944 10.1073/pnas.0807679106 PMC2664025

[B33] Fu N. Ji M. Rouard M. Yan H.-F. Ge X.-J. (2022). Comparative plastome analysis of musaceae and new insights into phylogenetic relationships. BMC Genomics 23, 223. doi: 10.1186/s12864-022-08454-3 35313810 PMC8939231

[B34] Gaut B. S. (2015). Evolution is an experiment: Assessing parallelism in crop domestication and experimental evolution: (Nei lecture, SMBE 2014, Puerto Rico). Mol. Biol. Evol. 32, 1661–1671. doi: 10.1093/molbev/msv105 26012904

[B35] Gonzalez-Segovia E. Pérez-Limon S. Cíntora-Martínez G. C. Guerrero-Zavala A. Janzen G. M. Hufford M. B. . (2019). Characterization of introgression from the teosinte zea mays ssp. mexicana to Mexican highland maize. PeerJ 7, e6815. doi: 10.7717/peerj.6815 31110920 PMC6501764

[B36] Green R. E. Krause J. Briggs A. W. Maricic T. Stenzel U. Kircher M. . (2010). A draft sequence of the neandertal genome. Science 328, 710–722. doi: 10.1126/science.1188021 20448178 PMC5100745

[B37] Guo Y.-L. Bechsgaard J. S. Slotte T. Neuffer B. Lascoux M. Weigel D. . (2009). Recent speciation of Capsella rubella from Capsella grandiflora, associated with loss of self-incompatibility and an extreme bottleneck. PNAS 106, 5246–5251. 19307580 10.1073/pnas.0808012106 PMC2659713

[B38] Häkkinen M. Langhe E. (2001). Musa acuminata in Northern Borneo: preliminary report. INIBAP, Montpellier, France. 23p.

[B39] Heslop-Harrison J. S. Schwarzacher T. (2007). Domestication, genomics and the future for banana. Ann. Bot. 100, 1073–1084. doi: 10.1093/aob/mcm191 17766312 PMC2759213

[B40] Hippolyte I. Jenny C. Gardes L. Bakry F. Rivallan R. Pomies V. . (2012). Foundation characteristics of edible musa triploids revealed from allelic distribution of SSR markers. Ann. Bot. 109, 937–951. doi: 10.1093/aob/mcs010 22323428 PMC3310492

[B41] Hope G. Kershaw A. P. Kaars S.v. d. Xiangjun S. Liew P.-M. Heusser L. E. . (2004). History of vegetation and habitat change in the austral-Asian region. Quaternary Int. 118–119, 103–126. doi: 10.1016/S1040-6182(03)00133-2

[B42] Houel C. Bounon R. Chaïb J. Guichard C. Péros J.-P. Bacilieri R. . (2010). Patterns of sequence polymorphism in the fleshless berry locus in cultivated and wild vitis vinifera accessions. BMC Plant Biol. 10, 284. doi: 10.1186/1471-2229-10-284 21176183 PMC3022909

[B43] Janssens S. B. Vandelook F. Langhe E. D. Verstraete B. Smets E. Vandenhouwe I. . (2016). Evolutionary dynamics and biogeography of musaceae reveal a correlation between the diversification of the banana family and the geological and climatic history of southeast Asia. New Phytol. 210, 1453–1465. doi: 10.1111/nph.13856 26832306 PMC5066818

[B44] Jeensae R. Kongsiri N. Fluch S. Burg K. Boonruangrod R. (2021). Cultivar specific gene pool may play an important role in musa acuminata colla evolution. Genet. Resour. Crop Evol 68, 1589–1601. doi: 10.1007/s10722-020-01088-y

[B45] Kallow S. Panis B. Vu D. T. Vu T. D. Paofa J. Mertens A. . (2021). Maximizing genetic representation in seed collections from populations of self and cross-pollinated banana wild relatives. BMC Plant Biol. 21 (1), 415. doi: 10.1186/s12870-021-03142-y 34503446 PMC8431884

[B46] Li H. Durbin R. (2010). Fast and accurate long-read alignment with burrows–wheeler transform. Bioinformatics 26, 589–595. doi: 10.1093/bioinformatics/btp698 20080505 PMC2828108

[B47] Martin M. (2011). Cutadapt removes adapter sequences from high-throughput sequencing reads. EMBnet.journal 17, 10–12. doi: 10.14806/ej.17.1.200

[B48] Martin G. Baurens F. Hervouet C. Salmon F. Delos J. Labadie K. . (2020a). Chromosome reciprocal translocations have accompanied subspecies evolution in bananas. Plant J. 104, 1698–1711. doi: 10.1111/tpj.15031 33067829 PMC7839431

[B49] Martin G. Cardi C. Sarah G. Ricci S. Jenny C. Fondi E. . (2020b). Genome ancestry mosaics reveal multiple and cryptic contributors to cultivated banana. Plant J. 102, 1008–1025. doi: 10.1111/tpj.14683 31930580 PMC7317953

[B50] Martin S. H. Davey J. W. Jiggins C. D. (2015). Evaluating the use of ABBA–BABA statistics to locate introgressed loci. Mol. Biol. Evol. 32, 244–257. doi: 10.1093/molbev/msu269 25246699 PMC4271521

[B51] McKenna A. Hanna M. Banks E. Sivachenko A. Cibulskis K. Kernytsky A. . (2010). The genome analysis toolkit: A MapReduce framework for analyzing next-generation DNA sequencing data. Genome Res. 20, 1297–1303. doi: 10.1101/gr.107524.110 20644199 PMC2928508

[B52] McKey D. Elias M. Pujol B. Duputié A. (2010). The evolutionary ecology of clonally propagated domesticated plants. New Phytol. 186, 318–332. doi: 10.1111/j.1469-8137.2010.03210.x 20202131

[B53] Meyer R. S. Purugganan M. D. (2013). Evolution of crop species: Genetics of domestication and diversification. Nat. Rev. Genet. 14, 840–852. doi: 10.1038/nrg3605 24240513

[B54] Miller A. J. Gross B. L. (2011). From forest to field: Perennial fruit crop domestication. Am. J. Bot. 98, 1389–1414. doi: 10.3732/ajb.1000522 21865506

[B55] Morris J. L. Puttick M. N. Clark J. W. Edwards D. Kenrick P. Pressel S. . (2018). The timescale of early land plant evolution. PNAS 115, E2274–E2283. 29463716 10.1073/pnas.1719588115 PMC5877938

[B56] Paradis E. Schliep K. (2019). Ape 5.0: An environment for modern phylogenetics and evolutionary analyses in r. Bioinformatics 35, 526–528. doi: 10.1093/bioinformatics/bty633 30016406

[B57] Perrier X. Jacquemoud-Collet J.-P. (2006). DARwin software. CIRAD. Available at: https://darwin.cirad.fr/ as stated on the software webpage: https://darwin.cirad.fr/feedback.php

[B58] Perrier X. Jenny C. Bakry F. Karamura D. Kitavi M. Dubois C. . (2019). East African Diploid and triploid bananas: A genetic complex transported from south-East Asia. Ann. Bot. 123, 19–36. doi: 10.1093/aob/mcy156 30247503 PMC6344093

[B59] Perrier X. Langhe E. D. Donohue M. Lentfer C. Vrydaghs L. Bakry F. . (2011). Multidisciplinary perspectives on banana (Musa spp.) domestication. PNAS 108, 11311–11318. doi: 10.1073/pnas.1102001108 21730145 PMC3136277

[B60] Pompidor N. Charron C. Hervouet C. Bocs S. Droc G. Rivallan R. . (2021). Three founding ancestral genomes involved in the origin of sugarcane. Ann. Bot. 127, 827–840. doi: 10.1093/aob/mcab008 33637991 PMC8103802

[B61] Pritchard J. K. Stephens M. Donnelly P. (2000). Inference of population structure using multilocus genotype data. Genetics 155, 945–959. 10835412 10.1093/genetics/155.2.945 PMC1461096

[B62] Pujol B. David P. McKey D. (2005). Microevolution in agricultural environments: how a traditional Amerindian farming practice favours heterozygosity in cassava (Manihot esculenta crantz, euphorbiaceae). Ecol. Lett. 8, 138–147. doi: 10.1111/j.1461-0248.2004.00708.x

[B63] Purugganan M. D. (2019). Evolutionary insights into the nature of plant domestication. Curr. Biol. 29, R705–R714. doi: 10.1016/j.cub.2019.05.053 31336092

[B64] Rambaut (2006-2016) FigTree v1.4.3: Tree figure drawing tool. Available at: http://tree.bio.ed.ac.uk/software/figtree.

[B65] Ross-Ibarra J. Morrell P. L. Gaut B. S. (2007). Plant domestication, a unique opportunity to identify the genetic basis of adaptation. PNAS 104, 8641–8648. doi: 10.1073/pnas.0700643104 17494757 PMC1876441

[B66] Rouard M. Droc G. Martin G. Sardos J. Hueber Y. Guignon V. . (2018). Three new genome assemblies support a rapid radiation in musa acuminata (Wild banana). Genome Biol. Evol. 10, 3129–3140. doi: 10.1093/gbe/evy227 30321324 PMC6282646

[B67] Rouard M. Sardos J. Sempéré G. Breton C. Guignon V. V. den Houwe I. . (2022). A digital catalog of high-density markers for banana germplasm collections. PLANTS PEOPLE PLANET, 4, 61–67. doi: 10.1002/ppp3.10187

[B68] Ruas M. Guignon V. Sempere G. Sardos J. Hueber Y. Duvergey H. . (2017). MGIS: Managing banana (Musa spp.) genetic resources information and high-throughput genotyping data. Database 2017. doi: 10.1093/database/bax046 PMC5502358 29220435

[B69] Santos J. D. Chebotarov D. McNally K. L. Bartholomé J. Droc G. Billot C. . (2019). Fine scale genomic signals of admixture and alien introgression among Asian rice landraces. Genome Biol. Evol. 11, 1358–1373. doi: 10.1093/gbe/evz084 31002105 PMC6499253

[B70] Sardos J. Christelová P. Čížková J. Paofa J. Sachter-Smith G. L. Janssens S. B. . (2018). Collection of new diversity of wild and cultivated bananas (Musa spp.) in the autonomous region of bougainville, Papua new Guinea. Genet. Resour. Crop Evol. 65, 2267–2286. doi: 10.1007/s10722-018-0690-x

[B71] Sardos J. McKey D. Duval M. F. Malapa R. Noyer J. L. Lebot V. (2008). Evolution of cassava (Manihot esculenta crantz) after recent introduction into a south pacific island system: The contribution of sex to the diversification of a clonally propagated crop. Genome 51, 912–921. doi: 10.1139/G08-080 18956024

[B72] Sardos J. Perrier X. Doležel J. Hřibová E. Christelová P. Van den houwe I. . (2016). DArT whole genome profiling provides insights on the evolution and taxonomy of edible banana (Musa spp.). Ann. Bot. 118, 1269–1278. doi: 10.1093/aob/mcw170 27590334 PMC5155597

[B73] Sempéré G. Pétel A. Rouard M. Frouin J. Hueber Y. De Bellis F. . (2019). Gigwa v2–extended and improved genotype investigator. GigaScience 8 (5), giz051. doi: 10.1093/gigascience/giz051 31077313 PMC6511067

[B74] Sharrock S. (1988). Report on the first IBPGR-QDPI banana germplasm collecting mission to Papua New Guinea, 27 February to 22 March 1988 (Rome, Italy: IBPGR), 47.

[B75] Sharrock S. Daniells J. W. Kambuou R. (1988). Report on the second IBPGR-QDPI banana germplasm collecting mission to Papua New Guinea, 22 October to 27 November 1988 (Rome, Italy: IBPGR), 29.

[B76] Sharrock S. Jones D. R. Banag J. (1989). Report on the third IBPGR-QDPI banana germplasm collecting mission to Papua New Guinea, 15 February to 12 March 1989 (Rome, Italy: IBPGR), 17.

[B77] Shigeta M. (1996). “Creating landrace diversity: The case of the ari people and ensete (Ensete ventricosum) in ethiopia,” in Redefining nature: ecology, culture and domestication (London: Routledge).

[B78] Simmonds N. W. (1956). Botanical results of the banana collecting expedition, 1954-5. Kew Bull. 11, 463–489. doi: 10.2307/4109131

[B79] Simmonds N. W. (1962)The evolution of the bananas. In: The evolution of the bananas. Available at: https://www.cabdirect.org/cabdirect/abstract/19630303919 (Accessed January 14, 2021).

[B80] Soltis P. S. Soltis D. E. (2009). The role of hybridization in plant speciation. Annu. Rev. Plant Biol. 60, 561–588. doi: 10.1146/annurev.arplant.043008.092039 19575590

[B81] Sun X. Jiao C. Schwaninger H. Chao C. T. Ma Y. Duan N. . (2020). Phased diploid genome assemblies and pan-genomes provide insights into the genetic history of apple domestication. Nat. Genet. 52, 1423–1432. doi: 10.1038/s41588-020-00723-9 33139952 PMC7728601

[B82] Sutanto A. Edison H. S. Amril R. Nasution F. Hermanto C. Cizkova J. . (2016). Collecting banana diversity in eastern Indonesia. Acta Hortic. 1114, 19–26. doi: 10.17660/ActaHortic.2016.1114.3

[B83] Turcotte M. M. Turley N. E. Johnson M. T. J. (2014). The impact of domestication on resistance to two generalist herbivores across 29 independent domestication events. New Phytol. 204, 671–681. doi: 10.1111/nph.12935 25039644

[B84] Varshney R. K. Thudi M. Roorkiwal M. He W. Upadhyaya H. D. Yang W. . (2019). Resequencing of 429 chickpea accessions from 45 countries provides insights into genome diversity, domestication and agronomic traits. Nat. Genet. 51, 857–864. doi: 10.1038/s41588-019-0401-3 31036963

[B85] Wu G. A. Terol J. Ibanez V. López-García A. Pérez-Román E. Borredá C. . (2018). Genomics of the origin and evolution of citrus. Nature 554, 311–316. doi: 10.1038/nature25447 29414943

