# Supplementary figures and images for: Hybridization, missing wild ancestors and the domestication of cultivated diploid bananas
Source: Front Plant Sci. 2022 Oct 7;13:969220. doi: 10.3389/fpls.2022.969220 (PMC9586208; doi:10.3389/fpls.2022.969220)

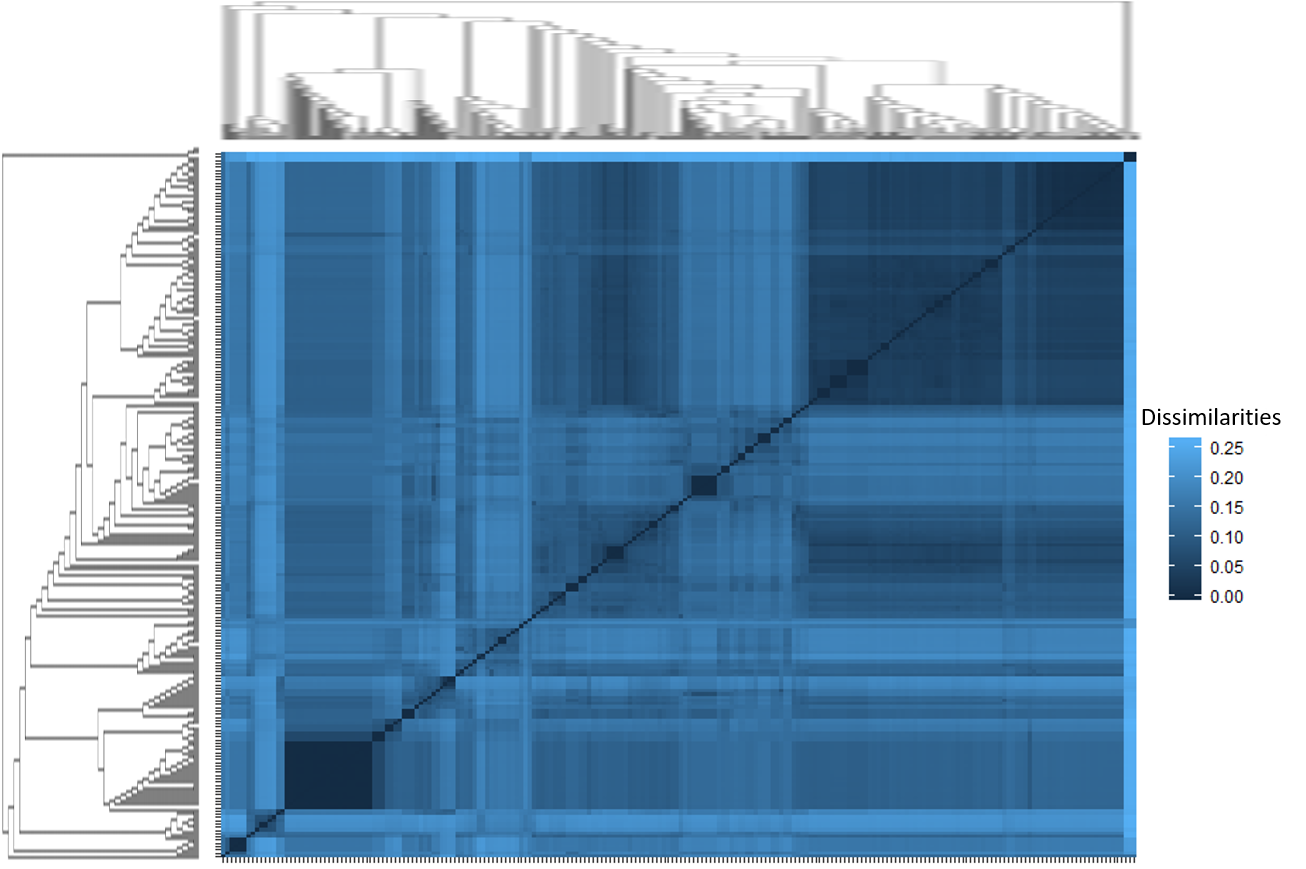

Supplement: Supplementary file 5 [file Image_1.png]
